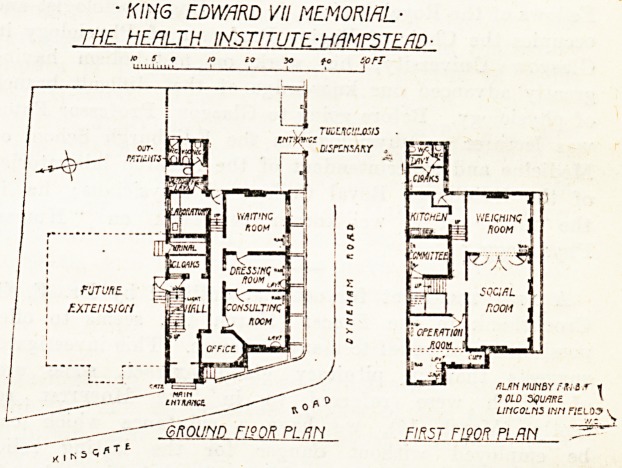# A Novel King Edward Memorial

**Published:** 1914-03-28

**Authors:** 


### HOSPITAL ARCHITECTURE AND CONSTRUCTION.

### A Novel King Edward Memorial.

The Borough of Hampstead can justly pride itself upon being one of the pioneers in social welfare and betterment schemes. It led the way in organising health societies in London; its own Council of Social Welfare is an institution which combines so many excellencies that it is difficult to do justice to them all. When the time came for Hampstead to consider what form its memorial to the late King should take, it was felt by those mainly responsible that no better memorial could be erected than a common home for the various municipal and voluntary bodies now actively dealing with social problems in the district. An influential committee was formed, a subscription list was opened, and, after energetic work and many difficulties, the new Health Institute was formally opened in December last. At present it is in full working, and is one of the most interesting social experiments in London, and, perhaps, also in the whole kingdom.

There has never been greater need in social work for co-operation and co-partnership between the various associations than there is at present. School medical inspection and the recent inception of an organised crusade against consumption, the National Insurance Act and the Labour Bureaus, the establishment of schools for mothercraft and domestic economy, and the various schemes for the treatment of physically defective children, have all been started on independent lines, and are all working, in most areas, with little regard to one another. This wasteful expenditure of time and trouble causes far greater overlapping than is necessary or desirable. The good done in one branch of work is lost in the transference to another department, which is independently organised and may be working on entirely different lines. There is no central control available, even in districts of small area. In school inspection this is especially the case. The school doctor follows up his children, but the process is necessarily incomplete; he does not know where they are treated; he cannot see them after they have left school, and he can hardly, therefore, fulfil his whole duty, or, in the words of Sir George Newman, "justify particular reasons of his existence in the State."

Hampstead has realised this fact; it has tried, by establishing this new Health Institute, to obtain at least a minimum of central control. The way in which it has proceeded is interesting as showing the diversity of the ramifications of social work it has had to deal with. Thus with regard to the important matter of the saving of infant life it has established an Infants' Health Committee, whose visiting work is supplemented by two Mothers' Clubs. This department is now housed in the Institute, where it has the use of a consulting room and a Baby Clinic. Next there is the Joint Tuberculosis Committee, of which the medical officer of health, the tuberculosis officer, and the school doctor are members. This department has annexed to it a modern and excellently equipped tuberculosis dispensary, with a separate approach, so that it can be isolated from the rest of the building. Thirdly, there is the care of the children. Here the Council of Social Welfare has relied mainly on the formation of Mutual Provident Aid Associations, generally with excellent results. By an arrangement with the Provident Dispensary doctors all child members are annually examined before they attain school-going age, when they pass automatically under the routine supervision of the school doctor. In this way the Council has successfully tackled the problem of the health of the child before school age. It has started a Supervisory Clinic and a Mothers' Club, both of which are housed in the Institute, where the dispensary doctors have the use of a fully equipped consulting and operating room, with a recovery and waiting room.

In addition the Institute provides, in its large and specially constructed basement, a commodious school of domestic economy, where free instruction is given to mothers and daughters; on its first floor a well-appointed social room, with annexed kitchen capable of catering for fifty persons. This room is to be used for first-aid and other classes, health lectures, parents' meetings, and in the evenings for evening lectures and socials, while on Sundays it is occupied in the morning by a men's adult school and in the evenings by the Gospel Temperance Mission.

Such institutes are too often so eminently practical; those responsible for their inception overlook the fact that attractiveness, cosiness, decoration, brightness, and comfort are very valuable educative factors in social work performed under such conditions. Here, however, they have been attended to. From an architectonic point of view the Institute is admirable. Two beautifully designed tiled fireplaces are in the social room, the gift of Mr. Nunn as a small tribute to the memory of the late Canon Barnett, whose ideals and aspirations have been an incitement to the founders of the Institute. On the staircase are some fine examples of Mr. Henry Holiday's panels; in the social room hang representative pictures by Collier and Parsons, lent for the service of the Institute by the respective artists. The furnishing is simple but well chosen; the rooms are bright and cheerful, with well-filled bookcases, flowers, and a few simple but decorative pieces of pottery.

There have arisen many memorials to the late King; few, one ventures to think, can have the extended range of usefulness of this simply-planned Institute, whose chief object is to promote close co-partnership between the various social agencies. Its potentialities, provided Hampstead recognises and uses them, are unlimited; its success should be an inducement for other boroughs to start co-operative schemes on a similar principle. For only by such co-operation can the uneconomical overlapping that is now so frequent in social work be obviated, as in the interests of the community it should be. Hampstead may be congratulated on having set up a model which is well worth imitating and one which it would be hard to surpass, except by a much more ambitious and costly design.

## Figures and Tables

**Figure f1:**